# PPR Control in a Sahelian Setting: What Vaccination Strategy for Mauritania?

**DOI:** 10.3389/fvets.2019.00242

**Published:** 2019-07-23

**Authors:** Ahmed Salem ElArbi, Yaghouba Kane, Raphaelle Metras, Pachka Hammami, Mamadou Ciss, Assane Beye, Renaud Lancelot, Adama Diallo, Andrea Apolloni

**Affiliations:** ^1^Ministère de l'Elevage/Mauritanie, Nouakchott, Mauritania; ^2^EISMV Dakar, Dakar, Senegal; ^3^CIRAD, UMR ASTRE, Montpellier, France; ^4^ASTRE, Univ Montpellier, CIRAD, INRA, Montpellier, France; ^5^Laboratoire National de l'Elevage et de Recherches Vétérinaires, Institut Sénégalais de Recherches Agricoles, Dakar, Senegal; ^6^FASEG, Université Cheikh Anta Diop, Dakar, Senegal

**Keywords:** PPR, West Africa, mathematical modeling, vaccination, cost-benefit analysis, global strategy for control and eradication (GSCE)

## Abstract

*Peste des Petits Ruminants* (PPR) is a viral disease affecting domestic and small wild ruminants. Endemic in large parts of the world, PPR causes severe damages to animal production and household economies. In 2015, FAO and OIE launched a global eradication program (GCSE) based on vaccination campaigns. The success of GCSE shall depend on the implementation of vaccination campaigns, accounting for husbandry practices, mobility and the periodicity of small ruminants' population renewal. In Mauritania, PPR outbreaks occur annually despite ongoing annual vaccination campaigns since 2008. Here, we developed a mathematical model to assess the impact of four vaccination strategies (including the GSCE one), the importance of their timing of implementation and the usefulness of individual animal identification on the reduction of PPR burden. The model was calibrated on data collected through *ad-hoc* surveys about demographic dynamics, disease impact, and national seroprevalence using Monte Carlo Markov Chain procedure. Numerical simulations were used to estimate the number of averted deaths over the next 12 years. The model results showed that the GSCE strategy prevented the largest number of deaths (9.2 million vs. 6.2 for random strategy) and provided one of the highest economic returns among all strategies (Benefit-Cost Ratio around 16 vs. 7 for random strategy). According to its current cost, identification would be a viable investment that could reduce the number of vaccine doses to distribute by 20–60%. Whilst the implementation of the identification system is crucial for PPR control, its success depends also on a coordinated approach at the regional level.

## Introduction

*Peste des Petits Ruminants* (PPR) is a viral infectious disease affecting domestic (goats and sheep) and small wild ruminants (1, 2). The virus can infect camels (3–5), cattle, and buffalos (3, 6) although their role in the transmission remains unclear. PPR virus (PPRV) is transmitted through close contact between infected and susceptible animals. Common signs of the infection are high fever, ocular and nasal discharges, erosive lesions on different mucous membranes, particularly in the mouth, diarrhea and respiratory distress. Because these symptoms are similar to those of other diseases such as rinderpest, pasteurellosis, and bluetongue (7), the clinical diagnosis is taken as provisional until confirmed by a laboratory. Depending on age and species (sheep are clinically more resistant than goats) the disease may be hyper-acute (mortality at 98% among 4–7 months old animals), acute (mortality at 60% among all population), mild (no mortality), or sub-clinical (8, 9). The sub-clinical form is frequent in Sahelian ruminants, in particular among sheep: the infected animal, although not showing any clinical signs, may shed the virus and transmit it to other animals by close contact (10).

Due to the severe impact of PPR on animal production, and following the successful rinderpest eradication, FAO and OIE have developed a strategy for PPR eradication by 2030 relying on vaccination campaigns and disease surveillance (11). Indeed, as for rinderpest, there are a very efficacious attenuated PPR vaccines that provides lifelong immunity and efficient PPR specific diagnostic tools for disease surveillance (12–15). Despite the similarities with rinderpest, the PPR eradication strategy should take account of some characteristics of small ruminant production that could hinder the process: the small ruminants population is much larger and grows faster than that of cattle; small ruminants have a lower socio-economic value and consequently less investments are made for their health; small ruminants can be sold more easily to cover household needs, and can be traded in large flocks (16). The PPR Global Strategy for the Control and Eradication (GSCE) is composed of 4 necessary steps: 1-Assessment, 2-Control, 3-Eradication, and 4-Post-eradication follow-up (11). In stage 2, mass vaccination (100%) of all animals older than 3 months of age is suggested in a first phase, followed by a phase of targeted vaccination of animals between 4 and 12 months of age. Previous work (16) has shown that, at worldwide level, the eradication programme would be highly beneficial economically, with an average benefit-cost ratio of 33.8, providing a compelling argument for PPR eradication. On the other hand, other works (17–19) showed that other costs, like the logistic (fuel for vehicles, maintenance of the cold chain etc.) and the personnel (time and missions to vaccinate animals) ones, and the vaccine wastage (doses given to already vaccinated animals) could have a relevant impact on the vaccination campaign, accounting for, in some cases, up to 70% of the campaign costs. To be effective, GSCE should be tailored to country epidemiological situation and take account of small ruminants production system dynamics.

Small ruminant production plays a major role in Mauritania economy. Indeed, goat and sheep production ensures (an almost) self-sufficiency for the country's red meat consumption and their trade represents a major source of income contributing to almost 70% of the agricultural GDP^1^. PPR is endemic in Mauritania, with outbreaks reported yearly during winter time (January-March) and during the Tabaski period^2^.

Livestock mobility and population turnover are two of the main factors contributing to the propagation and persistence of the virus in the Sahelian region (20, 21). In West Africa, animals, mainly adult ones, are moved in search of better grazing areas (i.e., transhumance) (21–23), to be sold alive at markets (i.e., for commercial reasons and at religious festivities such as *Tabaski*) (24, 25), or to be exchanged among families and relatives (i.e., *confiage*) (25, 26). Because of these movements, infected and susceptible (e.g., naïve) herds can get in contact, thus allowing virus transmission.

On the other hand, population renewal sustains endemicity of the virus. Depending on husbandry practices and agro-ecological systems, births are concentrated in 1 or 2 periods of the year. Newborn animals from mothers with PPR antibodies (i.e., naturally immunized or vaccinated), can inherit maternal antibodies, through colostrum, and be protected from the infection for the first 2–4 months of their life. After this period, animals become fully susceptible to PPR (27, 28) thus ensuring the regular re-introduction in the population of fully susceptible animals that could feed the disease cycle (29).

PPR represents a huge constraint to the development of Mauritania, affecting the economies of middle-low incomes families. Symptomatic animals are treated with antibiotics for a week (30) and vitamins. These treatments are done at the disease onset, aiming to prevent secondary bacterial infection, reduce severity of the disease and minimize economic losses. A retrospective study in Mauritania. El Arbi (10) reported that the practice of giving antibiotics to animals is widespread among herders and livestock owners, although this is not recommended by OIE. Vaccination remains the only viable and practical tool to control the disease as it will be impossible to implement drastic sanitary measures, stamping out policy and restriction of animal movements in Mauritania. Small ruminants vaccination campaigns against PPR are implemented since 2008 but the coverage rate remains low (ranging from 2 to 8% between 2008 and 2010; and in 2018 reaching 15.6% of the population) (10). The low vaccination coverage can be explained by several factors, such as: (i) vaccination is not compulsory, except in case of outbreaks; (ii) there is a lack of information about vaccine benefits (10), and most importantly, (iii) logistics issues, such as the cold chain for maintaining the vaccine, constrain the distribution of the vaccines. Nevertheless, for small ruminants' owners, vaccinating an animal costs 0.10 USD against 1.40 USD for giving antibiotics treatment (10).

To stop the epidemics spreading the GSCE (11) requires the post-vaccination immunity coverage to reach at least 70% (PVIR threshold), 80% to consider the country PPR-free. The PVIR threshold depends on the basic reproduction ratio *R*_0_ that could vary depending on the characteristics of the geographical area and epidemic setting. For example, the authors of Fournié et al. (31) estimated a lower value of the PVIR threshold, around 61.7% for the Ethiopian small ruminant population. Moreover, as shown in Hammami et al. (29, 32) for sub-Saharan Africa herds, the immunity coverage is strongly dependent on the month of vaccination.

Effective and efficient use of public funds is considered as necessary in the context of limited resource availability. In the Mauritanian context, the economic evaluation of PPR eradication scenario options through a cost-benefit analysis is therefore of great interest as it would inform the government about the most cost-effective choice, at community level, between financing of the vaccination campaign and the management of disease outbreaks by breeders. In this work, we used a dynamic model to estimate the impact of PPR in Mauritania and economic benefits of different vaccination strategies, some of them already being in place, others to be implemented, for the period 2018–2030. Based on recent epidemiological and socio-economic data collected on the field, our model takes account of both transmission and demographic dynamics of the Mauritanian national herd. Dynamical models are commonly used in human and animal health, and have been applied to study cost-effectiveness or cost benefits of vaccination strategies (33–35) mainly for their capacity of assessing indirect effects of vaccination (36, 37). A similar model was developed for the Ethiopian national herd (31). We also used the model to assess if and under which conditions identification and “identification and screening” could be viable procedures to reduce the number of vaccine doses to distribute by minimizing vaccination wastage.

## Materials and Methods

### Study Area

Mauritania is located on the African Atlantic coast, confining with Morocco, Western Sahara, Algeria, Mali, and Senegal. The northern part of the country is hyper arid, while the rest is arid (38). The country is divided in 15 Wilayas (i.e., regions), subdivided in 44 Moughataas (departments). Most of the population is concentrated along the coast, mainly in Nouakchott accounting for almost a quarter of the population, and along the river Senegal in the South. In 2016, Mauritanian small ruminant national population counts around 6.2 million goats and 9.6 million sheep (http://www.fao.org/faostat/), mostly located in the Eastern (50% of national herd) and Southern (35%) Wilayas along the Senegal River. According to the recent demographic survey done by ONARDEL^3^ and Mauritanian Veterinary Services, small ruminant herds are mixed (sheep and goats) with a higher proportion of sheep, reaching 70% of the herd.

The first documented occurrence of PPR dates back to 1982 in the Gorgol Wilaya (39). Since then, the disease has been considered endemic in the country. According to the 2010 PPR national serosurvey, conducted under the frame of the AU-Ibar project VACNADA (http://www.au-ibar.org/vacnada), the estimated seroprevalence rate among small ruminants was 39% (95% C.I. 37–41%) (40).

### Data

**Figure 1:**
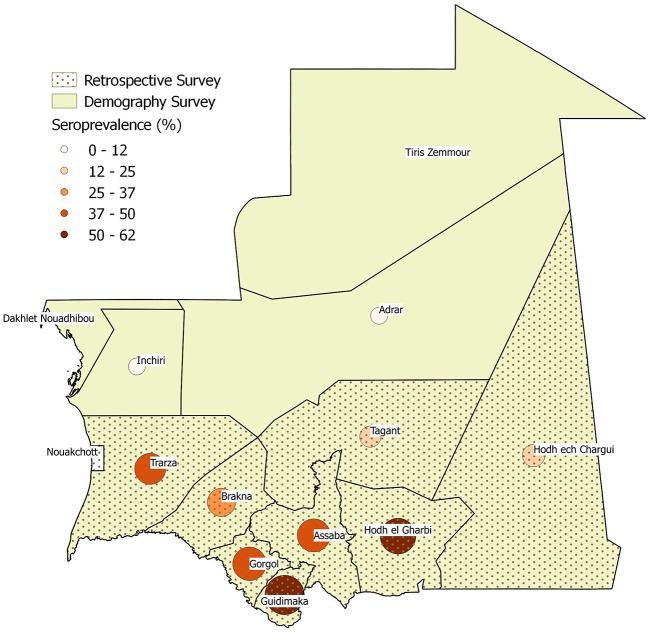
Wilayas included in the demographic (color), impact (dotted pattern) and seroprevalence (circles of darker color and increasing size by positive percentage) surveys.

Data on herd demography, PPR seroprevalence and disease impact were collected by ONARDEL officers through *ad-hoc* national surveys to calibrate the epidemiological model. Data on herd demography, which include deaths, births, purchases, and sales of small ruminants were collected in 2015 through a survey among all the Wilayas, except the Nouakchott district (investigated Wilayas are the colored ones in Figure 1). During the same year, a retrospective study was conducted to retrieve information on the impact of PPR outbreaks, number of cases and deaths, in the 12 previous months. The study was done in 10 Wilayas located in the three major pastoral areas of Mauritania: Hods, Assaba, and Senegal River valley (patterned Wilayas in Figure 1). Finally, in 2010 a national serosurvey campaign was conducted as part of the VACNADA project activities to estimate PPR prevalence in 10 Wilayas (circles in Figure 1).

#### Demographic Data

A total of 2,892 small ruminant herds were surveyed among 12 Wilayas in the pastoral area. Information were collected about herd size, their composition in terms of sex (male and female), species (goats and sheep) and age (weans, younger than 6 months, and animals older than 6 months), and the demographic events which had occurred over the previous 12 months (births and deaths, animal entry and exit, see Table 1).

**Table 1:** Data from national demographic survey conducted in 2015.

**Species**	**Weans** **(<6 months old)**	**Others**	**Population**	**Births** **(last 12 months)**	**Entries**	**Deaths** **(last 12 months)**	**Exit**
Goats	43,729	113,387	157,116	38,046	1,730	9,998	12,629
Sheep	287,869	107,975	395,844	97,814	7,114	36,673	43,973
Total	331,598	221,362	552,960	135,860	8,844	46,671	56,602

*Entries and exits indicate the total number of animals entering or leaving the herd, respectively, due to purchases, sales, festivities and loans*.

#### Serological Data

Sheep and goats older than 3 months and coming from 21 villages in 10 Wilayas were sampled for a total of 1,897 small ruminants (711 goats and 1,186 sheep). The collected sera were tested for the presence of IgG antibodies against PPRV. For each animal, information about species, sex, and age (based on teeth counting) were also collected. The results show a significant difference according to species, with sheep presenting a higher prevalence level than goats (Table 2).

**Table 2:** Number and percentage (in brackets) of seropositive PPR animals in each age group, by species.

	**Goats**		**Sheep**		**Small ruminants**	
**Age (month)**	**Population**	**Positive (%)**	**Population**	**Positive (%)**	**Population**	**Positive (%)**
3–6	16	6 (37.5)	34	9 (26.5)	50	15 (33.3)
6–12	136	44 (32.4)	233	102 (43.8)	369	146 (39.6)
12–24	156	58 (37.2)	285	117 (41.1)	441	175(39.7)
24–36	145	49 (33.8)	260	103 (39.6)	405	152 (37.5)
36–48	128	42 (32.8)	212	79 (37.3)	340	121 (35.6)
48+	130	44 (33.9)	162	90 (55.6)	292	134 (45.9)
Total	711	243 (34.2)	1186	500 (42.2)	1897	743 (39.2)

#### Disease Impact Data

Seven hundred and eight herders were surveyed using a semi-structured questionnaire over the events of the last 12 months, in particular: PPR knowledge; PPR cases and related deaths in the herd; intervention costs and the impact of the disease on the animal production, and epidemiological and economic data collected for more than 9,200 animals. Herders were chosen according to husbandry practices: transhumant or sedentary. No distinction was made between species in the premises, their gender and age. Table 3 reports some of the survey results that have been used to calibrate the model. The fatality rate has been evaluated as the ratio of PPR-related deaths over cases counted in a year.

**Table 3:** Summary table of disease impact survey by type of rearing.

	**Sedentary**	**Transhumant**	**Total**
Herds	178	530	708
Population (*n*)	7,645	89,570	97,215
PPR Cases (z)	1,147	7,167	8,314
PPR-related Deaths	459	1,792	2,251
Fatality rate	40%	25%	27.0%

### Model Structure and Calibration

**Figure 2:**
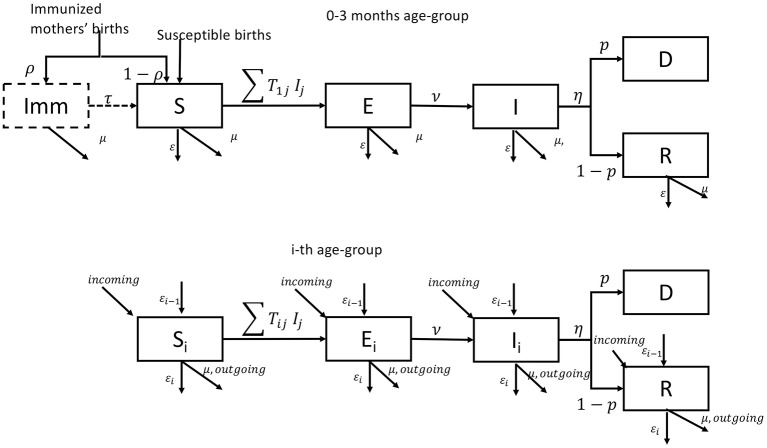
Pictorial representation of the epidemiological model for the first and i-th age group.

The small ruminant population (sheep and goats) demographics and transmission dynamics were simulated using a deterministic age-stratified compartmental model, without differentiating animals according to their species or their spatial location. A pictorial representation of the model is given in Figure 2 (to simplify, only the first and the i-th age-group are presented). The model considered a population stratified in seven age-groups (0–3 months-old, 3–6 months-old, 6–12 months-old, 12–24 months-old, 24–36 months-old, 36–48 months-old, and 48 months and older), the age structure being fixed a priori to match the age stratification of the serological survey. The youngest age-group (0–3 months old) accounts for the fact that a large fraction (around 92%) of newborn animals can be protected from the disease over the first 3 months of their life due to the potential inheritance of maternal antibodies against PPRV (27, 29). Because of this, and only for the first group, an extra compartment “Imm” is added to account for animals protected by maternal antibodies. In each age-group, susceptible animals (S) move to the latent state (E) after effective contacts with infectious animals (I), and subsequently become infectious (I). Infectious animals (I), after the infectious period, may recover (R) with a probability (1-p), or die for disease related causes (D) with a probability (p). The epidemiological dynamics is coupled with the underlying demographic one, with animals dying (with natural mortality rate μ), aging (with rate ε), leaving or entering the population due to trade exchanges to and from other countries (with rate *outgoing* and *incoming*), and reproducing (with rate α). We supposed that only animals older than 1 year could be exchanged and give birth. In Mauritania, births are concentrated in two specific moments of the year (August–September and December–January) and movements of small ruminants are concentrated in two periods: between April and June and around Tabaski (26) whose occurrence is anticipated every year of 11 days. The Tabaski-related peak of movements accounts for one fifth of the annual volume of animals traded and outbreaks are reported during this period. We adapted the transmission model to account for these characteristics. The list of parameters and their values is shown in Supplementary Table 2.

#### Demographic Parameters' Estimation

We considered a disease-free population, where the population in each age class (N_a_) was susceptible (no infected animal) to study the demographic dynamics of the population. At each time, each age class could change due to death, birth, aging, sale, and purchase of animals. We calibrated the model to estimate natural mortality, fertility, entry, and exit rates due to commercial exchanges. We supposed that the mortality rate was the same for all age groups (μ) except the last one (μ_5_). Fertility rate (α), entry (*incoming*), and exit (*outgoing*) ones were null for the first 3 age groups, and constant for all the other. The rates (α,μ,μ_5_, *incoming, outgoing*) were estimated by fitting model results to data in Table 1 using a Bayesian Framework (41). The model ran for a set of parameters to simulate the equivalent of 100 years, with a time step of 1 day. At the equilibrium, we estimated the proportion of deaths, births, entries and exits during the last 12 months, i.e.,

$$\begin{array}{l} p_{x}=\frac{x}{\sum_{a}N_{a}} \end{array}$$

Where *x* indicates the annual number of one of the events (death, birth, entry and exit) as simulated by our model. We sampled from the posterior distribution of the parameters (α, μ, μ_5_, *incoming, outgoing*) using Metropolis-Hastings algorithm, assuming uniform priors. The numbers of deaths, births, entry and exits (n_*x*_) reported in demographic survey data, Table 1, followed a binomial distribution.

$$\begin{array}{l} n_{x}\sim Binom\left(Population,p_{x}\right) \end{array}$$

Where *Population* is the number of small ruminants estimated during the survey. We ran 50 independent chains of 1,000 iterations. Results of the calibration procedure are provided in the Supplementary Material.

#### Transmission Model Calibration

Due to the age structure of the model, we introduced the transmission matrix *T* whose elements *T*_*ij*_ are the rate of transmission from infected animals of age group *j* to susceptible animals in age group *i* (42, 43). We imposed some transmission patterns, to reduce the number of parameters to estimate. Preliminary analysis of serological data showed that the percentage of seropositive steadily increases for the age-groups (3–6; 6–12 months) and subsequently flattens for older groups. This indicates that the force of infection (λ), the rate at which the susceptible population is infected changes drastically for animals younger and older than 1 year of age: the bulk of infections occurs among the youngest groups whilst new infections among the oldest groups seldom occur. We assumed that:

(1a,b) $$\{\begin{array}{c} T_{ij}=\beta_{0\;}\;i,j=1,2,3\\ T_{ij}=\beta_{1\;}\;i,j=4,5,6,7\; \end{array}$$

with β_1_ ≪ β_0_. Elements in Equations (1a) and (1b) are the matrix elements for the within-young (both i,j ≤ 3) and within-old (both i,j > 3) groups, respectively, and correspond to the block diagonal elements of the transmission matrix. The other elements of the transmission matrix, indicating transmissions between young (<1 year old) and old groups (>1 year old), we impose to be equal to one of the two (β_1_, β_0_). We also considered the case that the transmission parameter is constant across the age groups (β_1_ = β_0_).

Symptoms appear after 4–6 days (31, 44–46), so we considered an average latent period of 5 days, whilst death can occur after 5–10 days from the onset of symptoms (9, 44, 45) and we considered an infectious period of 5 days. The PPR-related mortality rate, or fatality rate (*p*), varies with age. Young animals (3–12 months old) are more likely to experience acute or super-acute infections with fatality rate ranging from 70 to 100% (31, 47, 48). Among older animals, more likely to experience a sub-acute form, the fatality rate is negligible, and was set between 0 and 2%. Through the calibration procedure, we estimated the values of the fatality rates for the youngest age-groups.

The transmission parameters β_1_, β_0_ and the fatality rates *p*_inf_, *p*_0_, for animals of age 0–3 months and 3–12 months, respectively, were estimated through calibration by fitting the model to serological and PPR-related death data in Tables 2, 3 and choosing the set of parameters minimizing the Deviance Information Criterion (DIC) value (49). To calibrate the transmission model, we consider that <1% of the population was initially infectious and let the system run for 100 years. At the equilibrium, we estimated, for each age-group the number of recovered animals (R_a_) and the total number of deaths caused by the infection in the last year (*D)* among the infected animals in the last year (*Z)*. Parameters estimation was done by fitting the fraction of the immune animals and the fraction of fatal cases to the serological data of Table 2 and the fatal cases of Table 3.

(2a,b) $$\{\begin{array}{c} \;p_{a}=R_{a}/N_{a}\\ p_{d}=D/Z \end{array}$$

where *N*_*a*_ is the population in the *a*-th age-group. The number of seropositives (*pos*_*a*_) and PPR-related deaths (*deaths*) follow a binominal distribution:

(3a,b) $$\{\begin{array}{c} pos_{a}=Bin(N_{a},p_{a})\\ deaths=Bin(Z,p_{d}) \end{array}$$

For all forms of the transition matrix T, we ran 50 Markov chains Monte Carlo (MCMC) Metropolis-Hastings algorithm of 1,000 iterations length and sampled from the posterior distribution of the parameters *Pars* = (β_1_, β_0_, *p*_0_, *p*_inf_). The best model had the lowest information criterion (DIC) (49) value. Results are shown in Supplementary Table 2.

### Forecasts and Impact of Four Different Vaccination Strategies

#### Baseline Case and 16 Tested Scenarios

We initially run the baseline scenario, where no vaccination is implemented. Then we considered 4 vaccination strategies that could be applied for the period 2019–2030:

- National Strategy (SR): half of the population is vaccinated. This is the current strategy implemented as a containment measure in Mauritania in case of appearance of new cases: only half of the animals of herds in the vicinity of outbreaks herd are vaccinated.
- Targeted scenario (ST): all animals between 4 and 12 months of age are vaccinated. This is the strategy planned for the next years in Mauritania.
- Mixed scenario (SM): National Strategy (SR) for the first 5 years, and targeted vaccination (ST) for the remaining years. This scenario has been introduced to take account of the delay for building up an identification system.
- Global Strategy for Control and Eradication (GSCE): all animals older than 3 months are vaccinated during the first 2 years, followed by a targeted vaccination (ST) of animals between 4 and 12 months until 2030. This is the procedure recommended by the Global Strategy (11) to be implemented for the first 4 years of the program.

For each strategy, the vaccination was implemented once a year but simulated on four different months of the year (March, July, October, and December), corresponding to specific demographic events (before transhumance, before the first peak of births, current period of vaccination, period around second peak of births) and periods of population renewal. Delayed vaccination could miss targeted population with long terms consequences on the population health status. In total, we simulated 16 different scenarios: 4 months of implementation for each of the 4 strategies. In all scenarios, vaccine is given to animals older than 3 months of age, since younger age animals might be protected by maternal antibodies and still have an immature immune system (11, 28). We also assumed that the vaccination is fully successful (all the animals vaccinated end up immunized) and confers a lifelong immunity. All scenarios start in 2018 with vaccination in October and 15.6% of animals vaccinated, according to information provided by Veterinary Service. We assessed the effectiveness of vaccination strategies by assuming that the higher the cumulative number of cases/deaths averted, the “better” the vaccination strategy. To further characterize the benefits of the different strategies, we introduced the notion of effective vaccine doses (*E*). Every year a certain quantity of vaccine doses (*Q*) is administrated to small ruminant population. Among them, due to the absence of an identification system, certain number of doses is given to already immunized animals either because they were previously vaccinated either because have already experienced the disease. We indicated these doses as wasted doses (*W*). The number of effective vaccine doses is the quantity of vaccine that is given to susceptible animals:

(4) $$\begin{array}{r} E=Q-W \end{array}$$

The quantity of effective doses varies according to the strategy but also depending on the period of the year and the health status of the population.

### Cost-Benefit Analysis

#### Disease-Related and Vaccination-Related Costs

Disease-related costs were distinguished from vaccination-related ones. The former consists of only treatment expenses (antibiotics + vitamins) for each infected animal, incurred by the owner. The treatment usually consists of antibiotics for 1 week and vitamins and, in average, it amounts at *c*_*tr*_ = 1.40$ for each infected animal. Consequently, the total disease-related cost is estimated as follow:

(5) $$\begin{array}{r} D=c_{tr}\;*\;Cases \end{array}$$

Two types of costs intervene in the cost of vaccination: the public and the private contribution. Administrating a dose of vaccine costs to the State *c*_*adm*_ = 0.3$ including the cost of the vaccine dose and logistic expenses, like the cold chain, equipment, personnel and carburant. Each herder pays a contribution of *c*_*pri*_ = 0.1$ for each animal vaccinated. Therefore, the vaccination-related costs can be estimated as follow:

(6) $$\begin{array}{r} V=\left(c_{adm}+c_{pri}\right)*\;Q=c_{V}\;*\;Q \end{array}$$

where *c*_*V*_ = (*c*_*adm*_+*c*_*pri*_) is the total cost associated to each vaccine dose.

A way to reduce vaccination costs is to reduce vaccine wastage (W), consequently administering only the effective number of doses (E). This can be achieved through individual identification and screening of animals: identification will avoid vaccinating animals that have been vaccinated during previous campaigns; whilst screening will allow to identify animals that have already experienced the disease and are already immunized. Excluding those animals from vaccination will reduce the required number of vaccine doses to administer the desired quantity of effective doses (E). In this analysis we are interested in assessing the viability and economical usefulness of the “identification and screening” procedure.

Identification and screening come with costs. We considered that animal identification is done during the vaccination. Consequently, identified animals are also vaccinated ones. As long as the cost of vaccinating all animals is higher than the one of identifying and vaccinated only un-identified animals, the identification is a viable cost:

(7) $$\begin{array}{r} cV{\;}^{*}\;Q\geq \left(c_{V}+c_{id}\right)*\left(Q-W\right) \end{array}$$

(8) $$\begin{array}{r} \frac{c_{id}}{c_{V}\;+\;c_{id}}\leq \frac{W}{Q} \end{array}$$

Where the left side of Equation (7) corresponds to the cost of vaccinating all the animals (identified and not) while the right side corresponds to the cost of vaccinating (and identifying) only the un-identified animals (Q-W).

**Figure 3:**
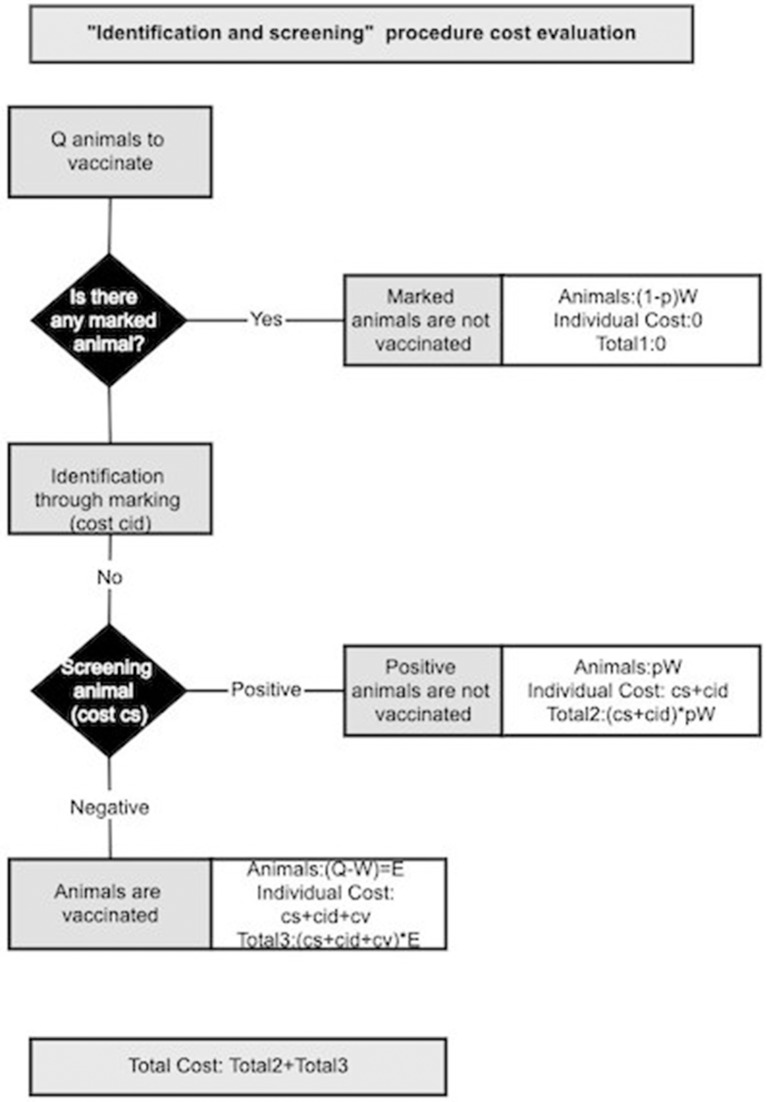
Schematic representation of the cost evaluation for “identification and screening” procedure.

Vaccine wastage can be further reduced by identifying and vaccinating only the susceptible animals among un-identified animals via an “identification and screening” procedure. We consider that during the vaccination campaign, animals are checked for identification marks: those already marked will not be vaccinated, whilst the un-marked ones will be marked and tested for the presence of PPR antibodies. Only those animals with negative results will be vaccinated. A pictorial representation for the “identification and screening procedure” is given in Figure 3. Animals excluded from vaccination are what we indicated with W, among them a fraction (1-p) is already marked, from previous vaccination, and a fraction p is seropositive. Besides the cost of dispensing doses, this procedure should account for the costs of identifying animals and screening. Some estimates of identification cost (*c*_*id*_) for animals are already available and we are interested in estimating the maximal acceptable cost for screening (*c*_*s*_). The “identification and screening” procedure is economically advantageous until its cost is < the cost of vaccinating all animals ($c_{V}*Q$). This condition can be expressed mathematically as:

(9) $$\begin{array}{r} c_{V}\;*\;Q\geq \left(c_{V}+c_{id}+c_{s}\right)\left(Q-W\right)+\left(c_{id}+c_{s}\right)pW \end{array}$$

(10) $$\begin{array}{r} c_{s}\leq \frac{c_{V}-\left(\left(c_{V}+c_{id}\right)*\left(1-W/Q\right)+c_{id}*p*W/Q\right)}{p*W/Q+\left(1-W/Q\right)} \end{array}$$

The maximal cost depends on the fraction of seropositive as well as the fraction of total wasted vaccine.

#### Benefits

The benefits evaluation involves two levels: the public and the private one. In the first case, benefits are mostly indirect: because of vaccination, less animals die from PPR and consequently animal production increases together with related products (milk, leather etc.). An increase in animal production means less importation and avoiding currency weakening. At the same time, improvement of household socio-economic conditions due to avoided mortality means higher taxes revenue. Private benefits could be both direct and indirect. Through vaccination, owners avoid medical expenses for treatments of infected animals. Furthermore, more animals, in better health, can be sold at markets increasing household income and providing some means to face emergency. Furthermore, a higher income could lead to improved social and health conditions of household members. A schematic summary of benefits is presented in Table 4.

**Table 4:** Summary of direct and indirect benefits from vaccination.

	**Direct**	**Indirect**
Public	N/A	• Improve food security by increasing animal production • Taxes return • Reduce import • Avoid currency weakening
Private	• Increase in animal production • Increase production of milk, dairy products, and leather • Higher revenue • Avoid medical expenses to treat infected animals	• Improve socio economic status • Improve health condition

In this analysis, we focus on direct benefits coming from the avoided losses due to PPR related deaths (BS) and avoided treatment expenses (BM) to assess the economic impact of the vaccination. The market values of young and old animals are different, with young ones, the more susceptible, sold at lower price than older ones (*r*_*young*_ < *r*_*adult*_). The BS can be estimated from the number of averted deaths in both groups as:

(11) $$\begin{array}{r} BS=r_{young}\;*\;YoungDeaths\text{\_}Averted\;+\;r_{adult}\;*\;AdultDeaths\text{\_}Averted \end{array}$$

Where *YoungDeaths*_*Averted* and *AdultDeaths*_*Averted* indicate the number of PPR-related deaths averted in the young and adult groups. The avoided treatment expenses (BM) can be estimated from the number of cases prevented as

(12) $$\begin{array}{r} BM=c_{tr}\;*\;\left(YoungCases\text{\_}Averted+AdultCases\text{\_}Averted\right) \end{array}$$

Where *YoungCases*_*Averted* and *AdultCases*_*Averted* indicate the number of PPR cases averted in the young and adult groups.

The total benefit can then be evaluated as:

(13) $$\begin{array}{r} BT=BS+BM \end{array}$$

All the analysis, simulations, calibrations and plots were done using the software R v 3.4.3 (50) and the packages deSolve (51), fitR (52), and ggplot (53).

## Results

### Calibration Results

Based on the lowest value of the DIC (49), the transmission matrix optimizing the fit of the model can be written as:

$$\begin{array}{l} T=\left(\begin{array}{c} \begin{array}{c} \begin{array}{c} \begin{array}{c} \begin{array}{c} \begin{array}{c} \begin{array}{ccc} \begin{array}{ccc} \begin{array}{ccc} \beta_{0} & \beta_{0} & \beta_{0} \end{array} & \beta_{1} & \beta_{1} \end{array} & \beta_{1} & \beta_{1} \end{array}\\ \begin{array}{ccc} \begin{array}{ccc} \begin{array}{ccc} \beta_{0} & \beta_{0} & \beta_{0} \end{array} & \beta_{1} & \beta_{1} \end{array} & \beta_{1} & \beta_{1} \end{array} \end{array}\\ \begin{array}{ccc} \begin{array}{ccc} \begin{array}{ccc} \beta_{0} & \beta_{0} & \beta_{0} \end{array} & \beta_{1} & \beta_{1} \end{array} & \beta_{1} & \beta_{1} \end{array} \end{array}\\ \begin{array}{ccc} \begin{array}{ccc} \begin{array}{ccc} \beta_{1} & \beta_{1} & \beta_{1} \end{array} & \beta_{1} & \beta_{1} \end{array} & \beta_{1} & \beta_{1} \end{array} \end{array}\\ \begin{array}{ccc} \begin{array}{ccc} \begin{array}{ccc} \beta_{1} & \beta_{1} & \beta_{1} \end{array} & \beta_{1} & \beta_{1} \end{array} & \beta_{1} & \beta_{1} \end{array} \end{array}\\ \begin{array}{ccc} \begin{array}{ccc} \begin{array}{ccc} \beta_{1} & \beta_{1} & \beta_{1} \end{array} & \beta_{1} & \beta_{1} \end{array} & \beta_{1} & \beta_{1} \end{array} \end{array}\\ \begin{array}{ccc} \begin{array}{ccc} \begin{array}{ccc} \beta_{1} & \beta_{1} & \beta_{1} \end{array} & \beta_{1} & \beta_{1} \end{array} & \beta_{1} & \beta_{1} \end{array} \end{array}\right)\; \end{array}$$

where β_1_ < β_0_.

**Figure 4:**
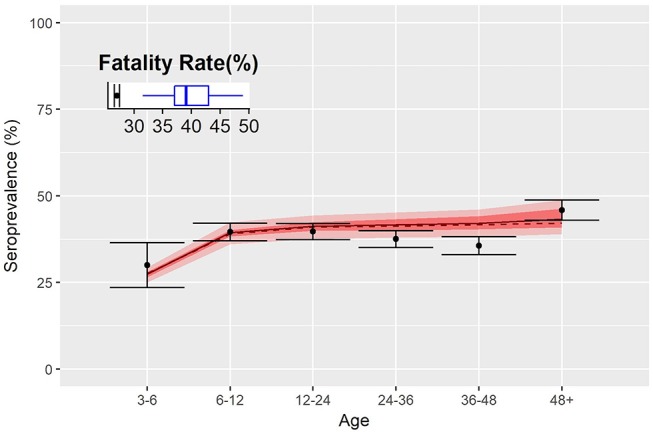
Fatality rates and seroprevalence estimated over the calibration of the transmission model. Dots represent the percentage of seropositive by age group, and shaded red area indicates 50 and 95% confidence interval of simulations. Inset shows the fatality rate estimate of the disease as from data (dots) and model (blue boxplot).

The fraction of immunized animals by age group, estimated by the model, matched well with sero-survey results (Figure 4) especially in the youngest and oldest groups. Nevertheless, the model predicts a slow increase of the seroprevalence with age, as expected for an endemic disease, whilst data show a decrease in the group between 3 and 4 years of age. The inset of Figure 4 shows the percentage of infected animals that subsequently die: dots correspond to the estimate of the fatality rate from data and the boxplot the estimates from the model. Despite the good agreement on the serological aspects, the model predicts a higher fatality rate relative to PPR (almost 10% above).

The calibration provided estimations for the demographic and epidemiological parameters. We sampled from the posterior distributions of the parameters to evaluate the basic reproductive ratio R_0_ using the Next Generation Approach as in Diekmann et al. (54). As reported in Supplementary Table 2, we found that the median value of *R*_0_ is around 2.9 (95% C.I. between 2.7 and 3.35). Consequently, the fraction of animals that should be vaccinated to reach the herd immunity threshold (*HIT* = 1 − 1/*R*_0_) is around 66% (95% C.I 64 and 71%).

### Vaccination Scenarios

We simulated the evolution of the disease from 2018 until 2030 considering an initial population composed of 15.8 million heads. We sampled from the parameter's distribution and for each combination, we ran the model for 100 years to estimate the equilibrium distribution of the population in the epidemiological compartments. The equilibrium distribution has been used as the initial state for all the simulation of the baseline (no vaccination) and vaccination scenario.

**Figure 5:**
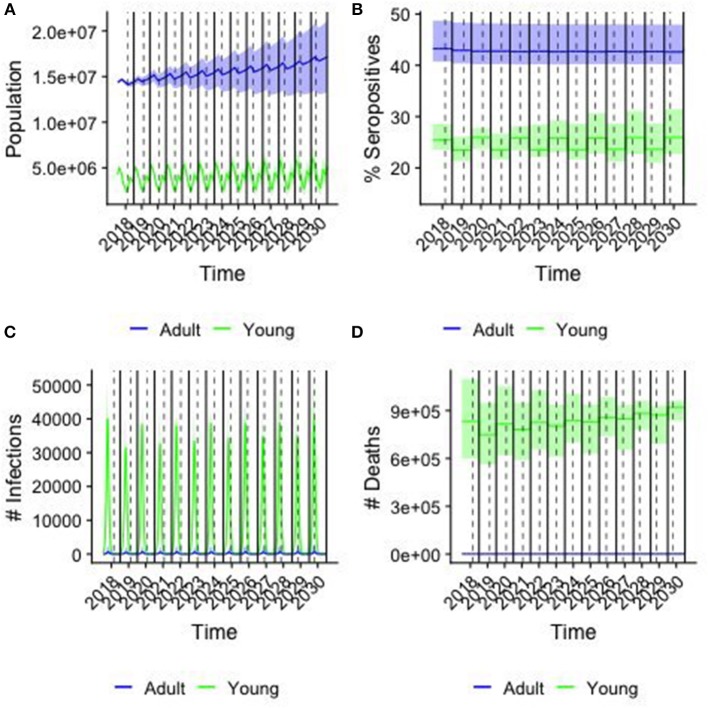
Results from the baseline case. colors correspond to young (<1 year old) and adults (>1 year old), solid line corresponding of the median and shaded area the 95% confidence interval of the simulations over a sample of 250 parameter values. Solid lines indicate the end/beginning of the year, while dashed line indicates Tabaski date. **(A)** The population in each age group by day **(B)** the percentage of seropositive animals in each group **(C)** the number of new infections by day **(D)** the cumulative number of PPR-related deaths by year.

Demographic and epidemiological results of simulations for the baseline scenario, i.e., when no vaccination is considered are illustrated in Figure 5, distinguishing animals of <1 year of age (young) from the older ones (adult). The daily trend of population is provided in Figure 5A, with peaks corresponding to the two birthing periods. Population grows over time, almost doubles in 10 years, following a trend similar to that predicted by FAOStat. Figure 5B shows the yearly average percentage of seropositive animals by age group. For adults the seroprevalence is constant (43%, C.I.[42.6,43.2]) along the years, while it is oscillating around the value (24.6 %, C.I[21,26.0]) for young. On average, the total seroprevalence is around 39.2% C.I[38.3,39.7]), comparable with the expected one from VACNADA data (10, 46). Figure 5C shows the daily number of new infections. The epidemics show a recurrent pattern with a peak of new infections occurring every year during the first few months, mostly among young animals. Finally, Figure 5D shows the year-cumulative number of PPR-related deaths. On average, every year, almost 2.5 million small ruminants would be affected by PPR and among them almost 8.5 × 10^5^ die of the disease. Few cases and deaths were registered in the adult class.

**Figure 6:**
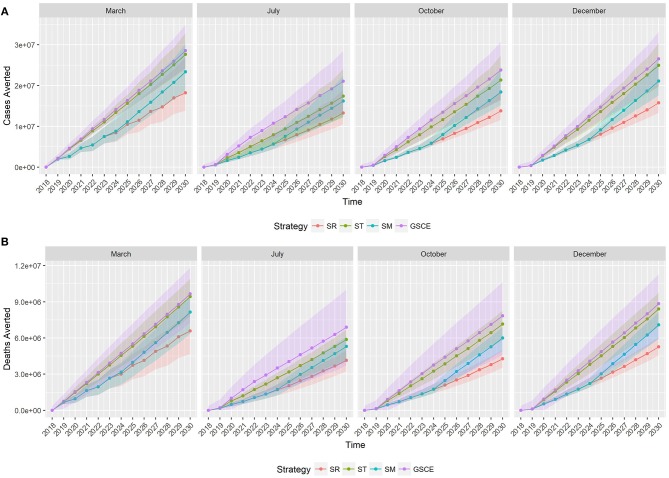
Median cumulative number of cases **(A)** and deaths **(B)** averted by vaccination. Colors correspond to the vaccination strategy, while line type to the month of vaccination. SR, National Strategy; ST, Targeted scenario; SM, Mixed scenario (SM) and GSCE, Global Strategy for Control and Eradication.

Figure 6 shows the cumulative number of infections (Figure 6A) and deaths (Figure 6B) averted along the 2019–2030 period by vaccination strategy (color) and month of vaccination (plot). In 2018, the number of cases and deaths averted is the same for all vaccination strategies, since we assumed the same quantity of vaccine was distributed for all scenarios, and accounted for around 2,700 infections and 650 deaths averted, on average. Starting from 2019, the effects of the different vaccination strategies are becoming distinct, and from 2020 we can see two different trends: on one side the GSCE and targeted (ST) strategies on the other the national strategy (SR) and the mixed one (SM). GSCE-vaccination appears to be the most effective strategy in terms of deaths and case reduction. The difference between targeted strategy (ST) and GSCE is essentially the vaccination coverage at the beginning, the latter considering the double of vaccine doses. As expected, the mixed strategy's (SM) effects become more evident with time. Since the first 5 years there is no difference from a random vaccination, whilst in the last few years the number of cases and deaths averted increases. The gap in cases and deaths averted between the SR and the GSCE strategy is that of a few million at the end (around 10 for the infections, and 3 for deaths). We notice that for each vaccination strategy there is a strong dependency on the month of vaccination, with vaccination done in March and December being the most effective ones.

**Figure 7:**
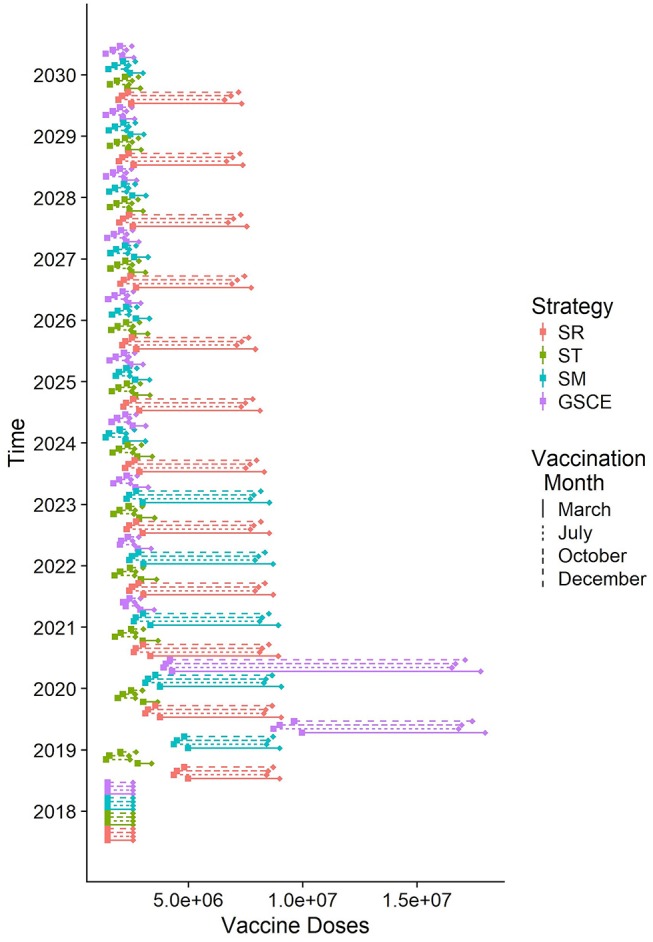
Quantity of vaccine distributed each year according to different strategies (median). For each line, on the right the total number of doses distributed, on the left the effective doses given to susceptible individuals. The length of the rod corresponds to the number of doses wasted because given to already infected individuals.

Targeted vaccination (ST) is the most effective in terms of doses distributed. In Figure 7 we report the quantity of vaccine distributed (Q) each year according to the different strategy and vaccination month, and the corresponding effective doses (E). Each year of the simulation (position on the y-axis), for each vaccination strategy (color) and month of implementation (type of line), we draw a segment whose ends correspond to the quantity of vaccine distributed (Q, right end) and the effective one (E, on the left). The segment's length quantifies the number of wasted doses (W). As can be easily seen the quantity of doses distributed each year by targeted and GSCE vaccination after 2020 is much smaller compared to a random allocation. However, for the first 2 years the quantity of vaccine allocated according to GSCE strategy is much higher than the others. Moreover, we notice that the quantity of vaccine wasted (W) is much smaller for targeted (ST) vaccination than any other vaccination strategy: less vaccine is wasted. For the first 2 years of implementation, the quantity of vaccine wasted for GSCE is higher than the other allocations and in 2020 the quantity of vaccine wasted by the GSCE strategy almost doubles. This is an effect of the previous mass vaccination campaign. According to our model, vaccination month has an effect on the quantity of vaccine to be distributed. For targeted vaccination (ST), for example, vaccination in March requires a slightly higher number of doses than for the other months. This is due to the presence of animals born during the second period of births, around December, which has become eligible to be vaccinated (older than 3 months).

### Costs and Benefits Analysis

**Figure 8:**
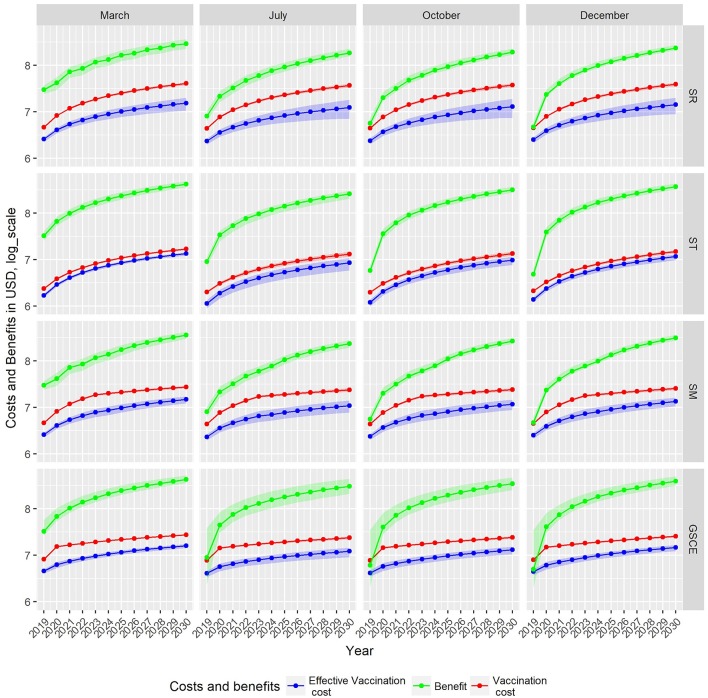
Cumulative cost and benefit for each strategy (row) and month of vaccination. Green indicates benefits from averted mortality, red the vaccination cost, and blue the loss due to wasted vaccination, all expressed in U.S. Dollars. Due to different scales of costs and benefit, estimates are given in Log_10_.

Figure 8 shows, for each scenario, the cumulative costs of the vaccination campaign (i.e., the cost of administrated? doses—red line), the costs of the effective vaccination (i.e., the cost of vaccinating only susceptible animals—blue line) and the total benefit from the averted death and averted treatment expenses (green line). For all scenarios, the estimated revenue is around one order of magnitude higher than the cost for vaccination. For SR strategies, independently of the month, the cost of vaccination campaign is always increasing. Except for vaccination implemented in March, for the SR case the benefits, during the first years, are comparable to vaccination costs. For ST strategies, effective and total vaccination costs (blue and red line, respectively) are comparable, mainly due to the fact that new born animals can be easily identified thus reducing vaccine wastage, and are in all cases lower than those of SR. The cumulative vaccination costs for ST monotonically increase indicating that an (almost) fixed quantity of vaccines is used every year. In the first 2 years, benefits suddenly increase. For the other two strategies (SM and GSCE), the vaccination costs are slightly higher than the targeted ones due to the massive vaccinations at the beginning. For SM and CSCE strategies implemented in months different from March, the benefits are comparable to the costs and steadily increase in the first 2 years. For all strategies, benefits from vaccination in March are immediately evident.

The total administration costs, the economic impact of vaccine wastage and the costs for the effective vaccination at the end of the period 2018–2030, together with the cumulative benefits were summarized in Table 5. We indicate with BCR the Benefit Cost Ratio, the amount of monetary gain realized by a single vaccination dose. The random (not targeted) strategies are the most expensive in terms of vaccine-administration costs and vaccine wastage: the more vaccine doses are distributed randomly, the more are wasted. Moreover, the benefits of SR strategies are the lowest among all the strategies. Targeted interventions, on the other hand, have the lowest administration costs and wastage, and, at the same time, the highest fraction of effective vaccination and economics benefit. The analysis of BCR shows that ST strategies are the most effective, whilst SR are the least ones. In Table 5, we identified in bold, for each strategy, the most effective month of vaccination. The effectiveness is estimated as the economic benefit by single dose distributed, as an example 1 USD invested in SR strategy in December returns 7.15 USD more. For all strategies, the most effective month of vaccination is March, except for the target one where it is December. In terms of BCR vaccinating in March is the most cost-effective period, except for ST, for whom December is the best period.

**Table 5:** Cumulative costs and benefits from the different vaccination scenario.

**Vaccination**		**Administration costs (million $)**				**Effective doses (million $)**				**Vaccination wasted (million $)**			
		**March**	**Jul**	**Oct**	**Dec**	**March**	**Jul**	**Oct**	**Dec**	**March**	**Jul**	**Oct**	**Dec**
	SR	40.5	36.7	37.6	39.0	21.7	18.4	19.1	20.4	18.8	18.3	18.5	18.6
	ST	16.9	13.1	13.4	14.9	14.3	9.7	10.6	12.5	2.6	3.4	2.8	2.4
	SM	27.6	23.7	24.1	25.6	18.1	14.1	14.8	16.5	9.5	9.6	9.3	9.1
	GSCE	27.3	23.7	24.1	25.5	18.9	15.0	15.9	17.5	8.4	8.7	8.2	8.0
**Benefits**		**BS (million $)**				**BM (million $)**				**BT = BS + BM (million $) (BCR = BT/cost)**			
		**March**	**Jul**	**Oct**	**Dec**	**March**	**Jul**	**Oct**	**Dec**	**March**	**Jul**	**Oct**	**Dec**
	SR	264	166	171	211	25.5	18.5	19.3	22.1	**289.9 (7.15)**	184.5 (5.0)	190.3 (5.1)	231.1 (5.9)
	ST	378	235	240	337	38.7	24.3	29.9	34.9	416.7 (24.6)	259.3 (19.4)	269.9 (20.1)	**371.9 (24.9)**
	SM	326	212	240	284	32.7	22.7	25.8	29.5	**358.7 (12.9)**	234.7 (9.9)	265.8 (11.02)	313.5 (12.2)
	GSCE	387	276	314	354	40.0	29.4	33.3	37.2	**427 (15.6)**	305.4 (12.9)	347.3 (14.4)	391.2 (15.3)

*SR, National Strategy; ST, Targeted scenario; SM, Mixed scenario (SM) and GSCE, Global Strategy for Control and Eradication. Top part of the table Vaccination information; bottom part benefits. Administration Costs indicate the total amount spent between 2018–2030, while Vaccination Wasted indicates the cost of the vaccine given to already vaccinated or immunized animals, with Effective doses we indicated the total costs of vaccinating only susceptible animals. Benefits are classified as those related to the market value of the animals (BS) and those related to the avoidance of medical treatments (BM). BT indicates the total benefit as the sum of the previous ones. Bold cells indicate those strategies with highest Benefit cost ratio (in parenthesis)*.

**Figure 9:**
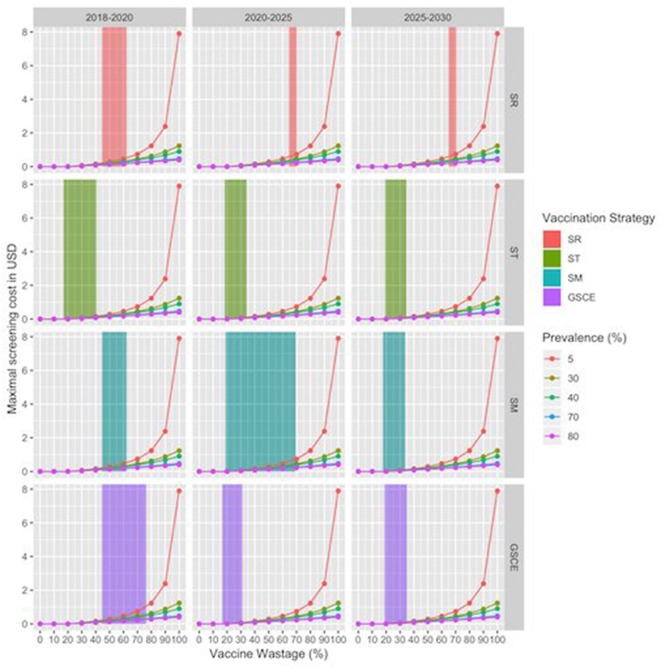
Maximal Screening cost per animal by vaccination strategy and period. X-axes correspond to the vaccine wastage, while y axis corresponds to maximal screening cost, zero value indicating that the procedure is not convenient. Line colors correspond to the evaluation for different sero-prevalence values. Shaded areas correspond to range of vaccination wastage in that period by vaccination strategy.

For SR strategies, the percentage of vaccines wasted is between 62 and 66 % depending on the month of vaccination, whilst for targeted strategies the figure lies between 20 and 35%, Reducing the wastage, through animal identification, could further increase the benefits. Identification cost (*c*_*id*_) amounts to 0.10$ per animal, thus contributing to 20% of the cost for vaccinating and identify animals (*c*_*id*_+ *c*_*V*_) that is, in most of the cases (not for the target strategies), less than the fraction of wastage. Finally, the estimated maximal cost for the screening procedure (*c*_*s*_) according to the scenario and percentage of seropositives in the population were presented in Figure 9. The maximal screening cost (*c*_*s*_) depends on the total vaccination cost (*c*_*V*_), the PPR prevalence and also the fraction of wastage. The latter two could change along the years. For each strategy we have considered three periods (2018–2020; 2020–2025; 2025–2030) to take account of possible variations in vaccine wastage. In Figure 9 each line corresponds to the maximal screening cost for a specific value of the prevalence, while the shaded areas correspond to the range of vaccination wastage in the period. Intersection between the line and the shaded area indicate the maximum affordable screening cost for the period. A negative or null value of (*c*_*s*_) indicates that identification screening procedure is not economically viable and then not worthy implementing. We notice that the higher the prevalence the lower is the maximal screening costs. For most of the strategy the maximal screening cost varies between 0 and 1 USD, except for the ST that is almost null. The maximal screening costs for strategy, decrease during the three periods, except for the SR strategy. In the late period for this strategy, the screening option is still viable till a cost of 1 USD. This is mainly due to the fact that SR strategy has the highest fraction of vaccine wasted. For the SM strategy, we notice that during the second period the vaccination wastage widely changes, due to switch from mass to targeted vaccination.

## Discussion

PPR is a major constraint to small ruminant production in Mauritania with serious negative impacts to the livelihoods of small farmer households. Several factors contribute to maintain the disease endemic in the area, among them population renewal and animal mobility. Safe and very effective vaccines are available for the control of the disease, which is now targeted for global eradication by year 2030. In this work, we presented a dynamical model for the transmission of PPR in Mauritania. Our model considers an undistinguished population of small ruminants, divided by age group, and takes account of some demographic factors (birth seasonality, movements, and renewal dynamics) ruling small ruminants' dynamics. Calibrated on serological data collected during the 2010 national serosurvey campaign and data from national surveys on herd's demography and disease impact, our model predicts a higher fatality rate for the disease than estimated from the data (37% against 27% from data). However, outbreaks investigations in three Wilayas during 2012 epidemics suggest a case-fatality rate close to our estimation (range [39; 58]%) (40, 46). This discrepancy could be related to the fact that estimation of PPR-related deaths wasn't confirmed by diagnostic control but based on surveyed recollection of previous year's PPR-related events. Due to this, the number of cases or the number of deaths related to PPR could have been easily miscalculated.

Our model predicts a value of *R*_0_ around 2.9, consequently the PVIR threshold is fixed around 66.6% a value in-between the GSCE PVIR estimate (70%) and those predicted by Fournié et al. (31).

In the baseline scenario, where no vaccination is applied, the model predicts around 16 million deaths due to PPR before 2030. Four different vaccination strategies have been considered and their implementation simulated in four different periods of the year. The use of a dynamical model allowed us to monitor the population distribution across the different epidemiological compartments at each time step of the simulation, but also to estimate the wastage of vaccine doses (W) due to re-vaccination of animals and vaccination of naturally immunized ones. Random strategies (SR) are the less effective in terms of the number of vaccine doses distributed (Q), wastage (W), and reduction of PPR-related deaths, whilst the GSCE strategies are, in the long term, the most effective in terms of cases and deaths reduction. Targeted strategies (ST) are the most convenient in terms of doses distributed, effectiveness of vaccination (higher ratio of effective vaccine doses), due to the targeting of young and probably non-immunized animals. On the other hand, the GSCE strategies, independently of the month, prevent the largest number of cases and deaths. The targeted strategies and the GSCE ones rely on the targeted vaccination of young animals, thus reducing the number of doses to distribute. As pointed out by Hammami et al. (32), it looks safer to implement at least 2 mass vaccination campaigns, firstly because GSCE strategy provides highest reduction in deaths and cases; secondly a mass vaccination could overcome the reticence of some herders. Strategies involved partial mass vaccination, like SM and SR, despite the large number of doses deemed less effective.

In terms of economic benefits, a GSCE strategy, independently of the vaccination month, has a much higher economic return compared to other strategies, whilst the target ones (ST) had the lowest costs associated. The cumulative vaccination costs for GSCE strategy, over the period 2018–2030, are higher than other strategies, mainly because of the mass campaign implemented at the beginning. Moreover, in this work we have compared only the cost of vaccination against those of administering antibiotics and vitamins, the common practice among herders. In the long term the abuse of antibiotics could lead to development of antimicrobial resistance with catastrophic consequences. Vaccination campaigns should be accompanied with sensibilization activities on the use/abuse of antibiotics.

The strategy choice and its implementation month have important effects on both costs and benefits at long term. For all strategies, vaccination should be implemented those months with highest presence of immunocompetent animals, i.e., animals older than 3 months of age and in good shape. In Mauritania these months correspond to the months of December and March. The end of March marks the beginning of the hot dry season, during which an animal's body and health conditions deteriorate, thus affecting their immune response, and herders begin leaving for transhumance. In our model, for all strategies, the best months for vaccinating animals are December and March. For vaccination implemented in these months the number of deaths and cases prevented and the BCR are the highest. Our model considers that vaccination is implemented in 1 month only, whilst Veterinary Service takes around 6 months to cover all the national territory (between October and April). BCR values for vaccination campaigns implement in the period December-March fluctuate between the two values reported in this article. Consequently, implementing a vaccination campaign in this period has the highest benefit. Mauritania encompasses several climatic areas (from hyper-arid in the North, to sub-humid in the South along the river Senegal) and demographic trends and transhumance's schedules depend on the natural resources available along the year, and could vary between years. To improve their efficacy and covering the largest fraction of animals, the vaccination schedule should take account of the resource availability in the different areas and prioritize those areas where resources could be depleted earlier (mainly from the north of the area). In our model we have considered that all neighboring countries have implemented the same vaccination strategy and the same vaccination coverage rate. Preliminary study, not reported in this article, has shown that vaccination coverage in neighboring countries could have a dramatic effect on Mauritania's national herd. We considered several scenarios in which the percentage of vaccinated animals among imported animals could vary from 0 (no vaccination) to 100% (all the animals are vaccinated). Focusing on the GSCE scenarios, if other countries are not implementing any vaccination campaign (vaccination coverage = 0%), the number of PPR-related deaths in Mauritania will be between 4 and 20% higher depending on vaccination month. On the other hand, when imported animals are all vaccinated (vaccination coverage = 100%) the number of deaths drastically reduces to almost 0. Reaching this level of vaccination coverage would be possible vaccinating animal at the border.

The BCR estimated for GSCE strategies in our model varies between 19.4 and 24.9 depending on the month of implementation. These values are far from those predicted by Jones et al. (16) (median 33.8 varying between 18.5 for low mortality area and 60.0 for high ones), with the benefit for treatment avoidance accounting for 10% of the total BCR. The discrepancies between our estimates and Jones' ones could be imputed to the small ruminants' market values used in our model.

Reducing the vaccine wastage could increase the economic benefits of vaccination. Vaccination wastage impacts the vaccination costs for a percentage varying from 20% (ST March) to 66.6% (SR in July). Wastage reduction can be achieved through the identification of the animals to avoid multiple vaccinations, a strategy recommended by FAO and OIE and sought to be implemented by Mauritania Veterinary Services. Our analysis provided strong arguments in favor of the identification procedure whose contribution to the final vaccination cost (administration + identification cost) is around 20%. Identification might then be considered as a viable option for all the strategies, in particular for the random ones. For targeted strategies (ST) the fraction of wasted vaccine is comparable to the contribution of identification to the total costs. However, due to the high BCR, the identification should be implemented. We also tested the possibility of adopting a two stage procedure, “identification and screening,” to increase the amount of effective vaccine doses and reduce the final number of animals to vaccinate. The possibility of deploying this type of procedure is hindered by many factors, among them the knowledge of the actual epidemiological situation, the type of vaccination strategy and the costs related to the screening test. The maximal cost for screening *c*_*s*_ depends on the prevalence and the vaccination strategy. A low value of *c*_*s*_ indicates that the implementation of the screening is not economically viable. In our cases we found that for most of our scenario the identification screening is not a viable solution. Moreover, depending on the particular test used, results couldn't be immediate thus complicating the vaccination procedure.

In our model, logistic costs were not detailed. However, considering that a single dose is 0.10 USD, almost 80% of the vaccine costs (0.40 USD) are related to logistic expenses. Mauritania geographical extension and the distribution of supporting infrastructure for maintaining the vaccine cold chain, constrain the number and the duration of field missions by Veterinary Services. The use of a thermostable vaccine could greatly reduce the logistic costs. Future studies should consider a detailed description of the logistic costs as part of costs benefits analysis of vaccination campaign as previously done for the Senegalese case (17).

Our model includes some characteristics of the Mauritania husbandry practices, like births and movement's seasonality. At one-year coarser temporal scale, the serological estimates are comparable to collected data. However, the model fails to predict the outbreaks occurring around Tabaski (reported by veterinarian services). These outbreaks are related to the rapid concentration of animals in urban areas, and the consequent burst of transmission. A model including multiple patches of population, linked by animal movements, could better describe the spatio-temporal patterns of disease propagation and reproduce the Tabaski peaks of infections.

In our model we have considered that GSCE strategy is applied all along the period 2018–2030 a period longer than suggested by OIE. Disrupting the vaccination could cause the re-insurgence of PPR, due to the re-introduction of the virus by transboundary movements, with catastrophic effects on small ruminants' production, as it has occurred in Morocco during 2016. Mauritanian commercial movements are mostly directed toward neighboring countries and import accounts for only 2% of the volume of animal traded. Moreover, due to the permeability of the borders and the lack of an integrated control and surveillance system in the area, PPR cannot be fully eradicated from Mauritania. In fact, because of these transboundary movements, infected and susceptible animals could be regularly introduced in Mauritania and re-ignite PPR outbreaks. The severity of these outbreaks would depend on the level of vaccination coverage of neighboring countries. Either Eradication requires a coordinated action at regional level with all countries in the region implementing vaccination policies aiming at covering 70–80% of the population of small ruminants and/or vaccinating imported animals at the border. In this case, this will mean adding extra vaccine doses, equivalent to the fraction of imported animals in Mauritania, for each vaccination campaign and create structures (vaccination parks) for the administration of the vaccine. Also, in this case to better assess the effect of vaccination in different countries a spatially structured model is required that takes in account the seasonality of mobility and the diffusion of immune animals.

In our model we have considered PPRV as the only pathogen circulating in the area and affecting small ruminant's production. However, other pathogens circulating in the region, like Pasteurellosis, could resurge after PPR eradication and disrupt the production chain. Because of this, PPR vaccination should be done during a joint campaign against other viruses and bacteria.

Results of our model suggests that vaccination campaigns done following the GSCE guidelines coupled with identification procedure could be an economically viable option to control PPR in Mauritania. However, eradication can be achieved only through a coordinate approach at regional level.

## Data Availability

All datasets analyzed for this study are included in the manuscript and the Supplementary Files.

## Author Contributions

AE, YK, and RL supervised the data collection and performed the preliminary data analysis. AA, RM, PH, and MC developed and implemented the dynamic model. AB, AA, and AE performed the cost-benefit analysis. All authors listed have made a substantial, direct and intellectual contribution to the work, and approved it for publication.

### Conflict of Interest Statement

The authors declare that the research was conducted in the absence of any commercial or financial relationships that could be construed as a potential conflict of interest.

## Footnotes

^1^GDP, Gross Domestic Product.

^2^Tabaski is the Muslim religious festivity of Aid-el-Kebir, during which each family sacrifices a sheep. The date of tabaski depends on the lunar calendar and every year is anticipated of 11 days.

^3^ONARDEL (Office National de Recherche et Développement de l'Elevage), it is the competent body for animal health in Mauritania
