# Supplementary material for: PPR Control in a Sahelian Setting: What Vaccination Strategy for Mauritania?
Source: Front Vet Sci. 2019 Jul 23;6:242. doi: 10.3389/fvets.2019.00242 (PMC6664874; doi:10.3389/fvets.2019.00242)
Supplement: Supplementary file 1 [file Data_Sheet_1.docx]

Supplementary Material

# Population dynamic model and estimation of demographic parameters

| 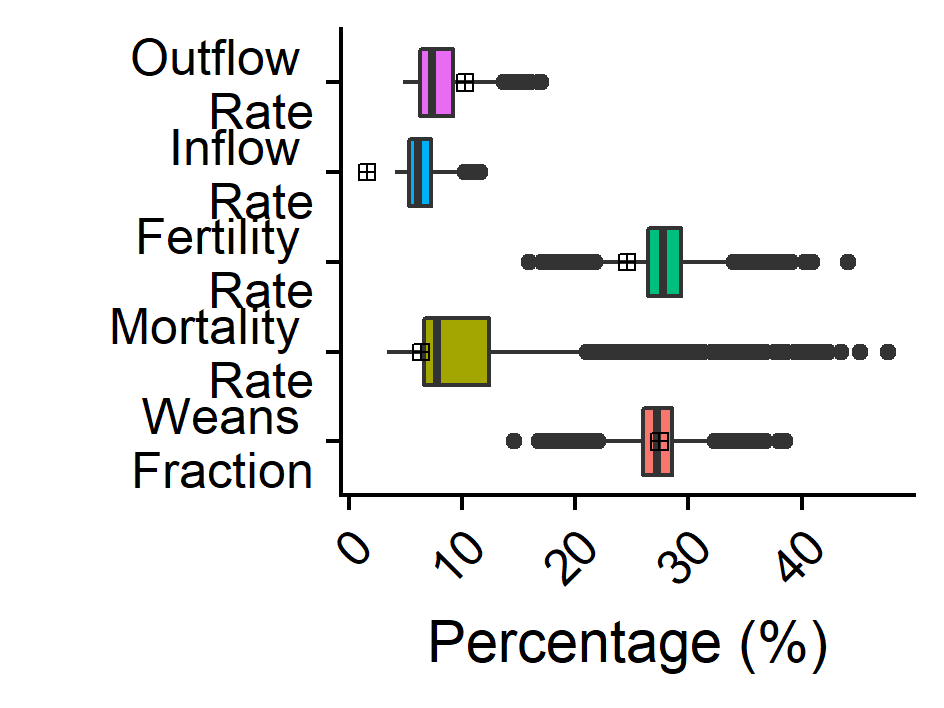 |
| --- |

Supplementary Figure 1. The weans' fraction, the number of deaths, births, entry and exit rate over the total population in the last year. Square corresponds to data and boxplot to simulations’ results.

# Catalytic model and estimation of the force of infection

| 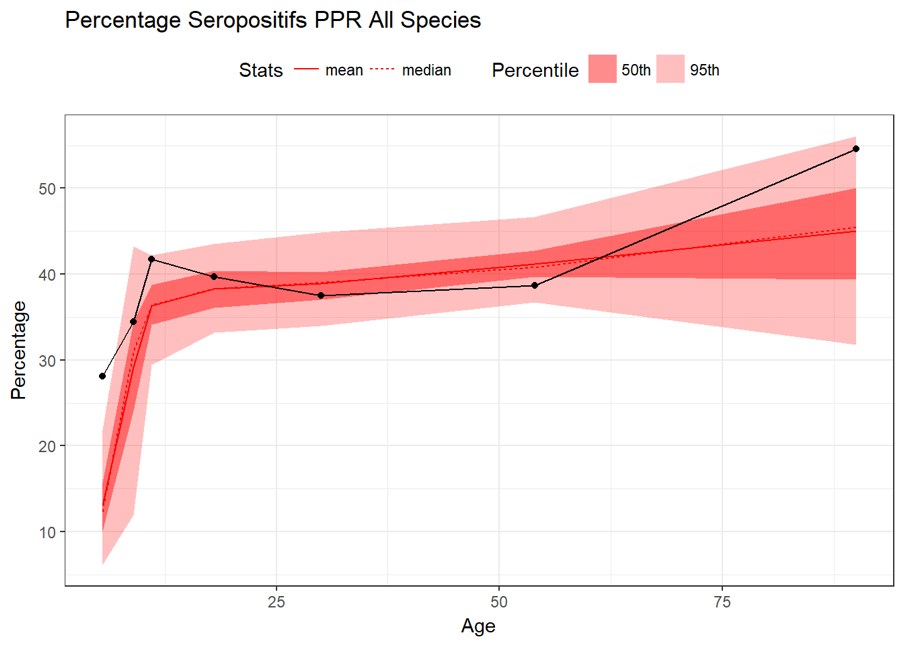 |
| --- |

Supplementary Figure 2. Results from the calibration procedure. Black line corresponds to the seroprevalence by age group, Red lines the mean and median of estimation by model. Shaded area 50 and 95% C.I.

Supplementary Table 1. Parameters’ estimation from MCMC procedure

| Species | Variable | Value ( 95% CI) |
| --- | --- | --- |
| Small Ruminants | $\lambda_{0}$ | 0.0019[0.0016,0.0020] |
|  | $\lambda_{1}$ | 5.1 e-05 [1.7e-05, 9.6e-05] |
|  | $a_{0}$ | 11.1 [10.4,12.4] |

# Parameters’ list and values

Supplementary Table 2 summarizes the list of parameters used in the model. All demographic parameters were estimated through calibration of disease-free/demographic model, while few of the epidemiological parameters were taken from literature or by expert opinion. The rest of the parameters were estimated through the calibration of the transmission model. In the table Source indicates the method used to evaluate parameter’s value or its source: *Calibration* indicates that parameters’ value has been estimated through fitting procedure; *Literature* indicates values extracted from literature; *Fixed* indicates values relative to the model structure; *Ministry* indicates that estimates come from the Mauritania’s Ministry of Agriculture and Animal Production

Supplementary Table 2. Model parameters. Parameters have been extracted from literature, provided by the Ministry, estimated through calibration procedure, or fixed by the model structure. All the parameters are daily rate.

|  | Notation | Variable | Values | Source |
| --- | --- | --- | --- | --- |
| Demographic parameters | µ | Natural mortality younger groups (0-48 months) | 0.00012 [2.0e-5,0.0015] | Calibration |
|  | μ_5_ | Natural mortality oldest group (>48 months) | 0.00094 [7.6e-5,0.0030] | Calibration |
|  | α | Birth rate | 0.00076 [0.0006,0.0010] | Calibration |
|  | Incoming | Entry rate | 4.3e-5 [2.3e-5,6.5e-5] | Calibration |
|  | Outgoing | Exit rate | 0.00021 [0.00015,0.00034] | Calibration |
|  | ε | Aging rate | 1/90 (0-3 and 3-6 months old) | Fixed |
|  |  |  | 1/180 (6-12 month) |  |
|  |  |  | 1/365 >12 months |  |
| Epidemiological parameters | ρ | Fraction of young with passive immunity (*i.e.* maternal antibodies) | 0.92 | Literature(28,30) |
|  | 1/τ | Passive immunity wane time | 90 days | Literature(30) |
|  | 1/ν | Incubation period | 5 days [4;6] | Literature (10,43,44) |
|  | 1/η | Infectious period | 5 days [3;7] | Literature (8,10,32,43) |
|  | p_1_ | Fatality risk for adult | 0.01 [0.0,0.02] | Fixed |
|  | β_0_ | Transmission parameter younger group (0-12 months) | 1.61 [1.50,1.82] | Calibration |
|  | β_1_ | Transmission parameter older group (>12 months) | 0.009[0.003,0.027] | Calibration |
|  | p_inf_ | Fatality risk 0-3 months | 0.15[0.013;0.26] | Calibration |
|  | p_0_ | Fatality risk 3-12 months | 0.79[0.72;0.87] | Calibration |
|  | R_0_ | Basic reproductive Ratio | 2.93[2.74;3.34] | Calibration |
| Cost-benefit parameters | c_adm_ | Cost vaccine dose administration (dose +logistic) | 0.3 $ | Ministry |
|  | c_priv_ | Contribution of owner to vaccination | 0.1 $ |  |
|  | c_id_ | Identification cost | 0.1 $ |  |
|  | c_tr_ | Treatment cost | 1.4 $ |  |
|  | r_young_ | Young (<1 year old) Animal  Market cost | 40 $ |  |
|  | r_adults_ | Adult Animal (>1 year old)  Market cost | 73 $ |  |
